# Associations between Cadmium Exposure and Taste and Smell Dysfunction: Results from the National Health and Nutrition Examination Survey (NHANES), 2011–2014

**DOI:** 10.3390/ijerph17030943

**Published:** 2020-02-03

**Authors:** Yi Zheng, Yun Shen, Zheng Zhu, Hui Hu

**Affiliations:** 1 Department of Epidemiology, College of Public Health and Health Professions and College of Medicine, University of Florida, Gainesville, FL 32610, USA; arieszheng@ufl.edu; 2 Department of Pharmaceutical Outcomes and Policy, College of Pharmacy, University of Florida, Gainesville, FL 32610, USA; yunshen@ufl.edu; 3 School of Nursing, Fudan University, Shanghai 200433, China; zhengzhu@fudan.edu.cn

**Keywords:** Cadmium, taste dysfunction, smell dysfunction

## Abstract

Background: Cadmium is a ubiquitous environmental pollutant and has been associated with many adverse health outcomes. However, little is known about the effect of cadmium exposure on taste and smell dysfunction. Methods: We used the National Health and Nutrition Examination Survey (NHANES) 2011–2014 to investigate the associations between blood cadmium and taste and smell dysfunction among 5038 adults aged 40–80 years old. Taste and smell dysfunction were defined by questionnaires, examinations, or both criteria. Results: In survey weighted logistic regression models adjusting for age, gender, race/ethnicity, income-to-poverty ratio (IPR), and education, individuals with a blood cadmium level in the highest tertiles had significantly higher odds of having perceived smell dysfunction (odds ratio (OR) = 1.41, 95% confidence interval (CI): 1.08, 1.84), perceived taste dysfunction (OR = 1.48, 95% CI: 1.16, 1.89), and taste dysfunction defined by both self-reported and objectively measured data (OR = 1.46, 95% CI: 1.03, 2.07). After further adjusting for body mass index (BMI), cigarette smoking, and alcohol drinking, consistent results were observed for perceived taste dysfunction (OR = 1.49, 95% CI: 1.10, 2.00), and no significant associations were found between cadmium exposure and other outcomes. Conclusions: Our findings suggest that cadmium exposure is associated with perceived taste dysfunction.

## 1. Introduction

Chemosenses, including taste and smell, enable us to identify food flavors and detect odors from surroundings and protect us from potential hazards such as bad food and leaking gases [1]. Loss of the ability to taste and smell has a direct impact on safety and quality of life [2]. For example, plenty of recent reports suggest that individuals with taste and smell disorders are more likely to have depressive symptoms [3,4,5,6]. A recent study reported potentially decreased sexual desire among subjects with smell impairment and depressive symptoms [7]. Taste and smell dysfunction is more prevalent among women and older adults [8,9,10]. Moreover, taste and smell dysfunction has been associated with many other conditions, such as substance use, multiple sclerosis, Alzheimer’s disease, Parkinson’s disease, and cancer [11,12,13,14,15,16,17]. Although many risk factors of taste and smell disorders have been examined [18,19], only a few studies focused on environmental pollutants [20]. Recently, Adams et al. [21] found that exposure to nitrogen dioxide was associated with smell dysfunction as well as other nasal diseases. However, little is known about the associations between exposure to heavy metals and taste and smell dysfunction.

Cadmium is a widely distributed environmental pollutant which has been associated with various negative health outcomes, including hypertension [22], renal cancer [23], prostate cancer [24], and cognitive function [25]. Major sources of cadmium exposure in the environment are soil, waste water, vegetables, and tobacco [26,27,28,29]. Although declines in cadmium exposure among teens and adults were observed in prior studies, long-term exposure to low levels of cadmium still poses risks of adverse outcomes to the general population [30]. Lee et al. [31] designed a cross-sectional study to investigate the risk of smell dysfunction in workers with occupational exposure to heavy metals such as cadmium and lead in Korea. Workers who were exposed to occupational pollutants were observed to have higher prevalence of smell dysfunction than those who were not. Most of the previous studies only assessed smell dysfunction using self-reported data. The potential association between cadmium exposure and taste and smell dysfunction among the U.S. population has not been well studied. To fill this gap, we conducted an analysis on the association between cadmium exposure assessed by blood cadmium concentration and taste and smell dysfunction measured by both self-reported and objective measures in a nationally representative sample.

## 2. Materials and Methods

### 2.1. Study Population

The National Health and Nutrition Examination Survey (NHANES) collects data from nationally representative samples in the United States through interviews, examinations, and laboratory tests using a multistage probability sampling design. We obtained data from the 2011–2014 NHANES, with 27,763 persons screened, 19,931 (71.8%) of them interviewed, and 19,151 (69.0%) of them examined in a Mobile Examination Center (MEC). Blood cadmium was measured in a subsample of individuals 1 year or older. Taste and smell questionnaire data were collected in a subsample of adults 40 years or older. We excluded individuals who were pregnant or breastfeeding at the time of interview (*n* = 85). Individuals with missing information on blood cadmium or self-reported taste and smell disorders were excluded. A total of 5038 adults between 40 and 80 years old were finally included in the analysis of self-reported taste and smell disorders. We also obtained taste and smell examination data from 3111 and 3516 individuals, respectively. These data were only available in 2013–2014. For the analysis on taste dysfunction using the taste examination data, we further excluded individuals who failed to tell the light intensity on the generalized Labeled Magnitude Scale (gLMS) in a correct rank (*n* = 280) and those who were allergic to quinine (*n* = 134). Individuals who had fair or poor understanding of the tests or with missing information on objectively measured outcomes were also excluded. Finally, a total of 1696 and 1502 individuals were included in the analysis using smell and taste examination data, respectively.

### 2.2. Perceived Taste and Smell Dysfunction

Information on self-reported taste and smell dysfunction was collected through interviews. Based on the validity and reliability of the taste and smell questionnaire [32], the perceived smell and taste dysfunctions were determined based on responses to two questions related to smell ability and six questions related to taste ability. Individuals who (1) had problems with smell in the past 12 months or (2) had worse ability to smell since age 25 were considered to have perceived smell dysfunction. Individuals were determined as having perceived taste dysfunction if they (1) had problems with taste in the past 12 months; (2) had worse ability to taste salty, (3) sour, (4) sweet, or (5) bitter since age 25; or (6) had persistent taste in mouth in the past 12 months.

### 2.3. Objectively Measured Taste and Smell Dysfunction

We also obtained data from the taste and smell examinations. The Modified Pocket Smell Test (M-PST, also known as the 8-item “scratch and sniff” test) was used to assess smell function based on the 40-item University of Pennsylvania Smell Identification Test (UPSIT) [33]. Eight specific odorants were released by scratching the test strips and presented to the individuals in a fixed order: chocolate, strawberry, smoke, leather, soap, grape, onion, and natural gas. For each odorant, individuals were asked to identify the presented scent by choosing one answer from the four listed alternative options. Individuals who were unable to correctly identify six or more odorants were categorized as having smell dysfunction.

Taste examinations were conducted using the 1 mM quinine (bitter) and 1 M NaCl (salty) tongue tip tests, as well as two different sequences of 0.32 M NaCl, 1 M NaCl, and 1 mM quinine whole-mouth taste tests. Individuals were asked to taste and rate the intensity of each given solution among individuals who passed the gLMS [34]. Based on previous reliability studies [32], taste dysfunction was determined using data from the 1mM quinine whole-mouth taste test, which had the highest intraclass correlation coefficient. Individuals who failed to correctly identify the bitter flavor of quinine in the whole-mouth taste test were determined to have taste dysfunction.

### 2.4. Blood Cadmium Measurement

Blood concentrations of cadmium were measured using whole-blood specimens from individuals aged 1 year or older. Samples were processed and stored under appropriate frozen (−30 °C) conditions and then transferred to the National Center for Environmental Health for laboratory testing. Whole-blood cadmium (μg/L) concentration was determined by inductively coupled plasma mass spectrometry. No changes existed between the two data cycles regarding equipment, laboratory methods, or facilities. Individuals with a blood cadmium concentration below the lower limit of detection (LLOD, 0.16 μg/L in 2011–2012 and 0.10 μg/L in 2013–2014) were assigned a value of LLOD divided by the square root of 2.

### 2.5. Covariates

Potential confounders were considered and selected based on directed acyclic graphs (DAGs) as shown in Supplemental Figure S1. Demographic characteristics including age (40–49, 50–59, 60–69, and 70–80), gender, race/ethnicity (non-Hispanic White, non-Hispanic Black, Hispanic, and others), education (<high school, high school, >high school), and income-to-poverty ratio (IPR: <1.0, 1.0–2.0, ≥2.0) were included. Body mass index (BMI) was categorized as underweight, normal, overweight, and obese. Cigarette smoking and alcohol drinking were categorized to three-level categorical variables (current, former, never).

### 2.6. Statistical Analysis

Descriptive statistics including weighted percentages with their 95% confidence intervals (CIs) were calculated. Blood concentrations of cadmium were log-transformed to account for their skewed distributions. Additionally, we categorized blood cadmium concentrations into tertiles. Weighted logistic regression models were used to assess the associations between cadmium exposure and taste and smell dysfunction, and odds ratios (ORs) and 95% CIs were calculated. Two minimal sets of covariates were adjusted based on the DAGs (Supplemental Figure S1) with different causal assumptions. The crude-adjusted models controlled for age, gender, race/ethnicity, IPR, and education. In the fully adjusted models, we additionally controlled for BMI, smoking status, and alcohol drinking. Both models were constructed for self-reported outcomes, objectively measured outcomes, and codefined outcomes (taste and smell dysfunction defined by both self-reported and examination data). A 2-year MEC exam weight was used for analyzing objectively measured data, and a 4-year subsample weight was used for self-reported data. All analyses were conducted using the “survey” package in R (3.5.1, R Core Team, Vienna, Austria).

## 3. Results

Among the 5038 individuals aged 40–80 years old included in the analyses of self-reported taste and smell dysfunction, 907 (18.0%) and 771 (15.4%) of them reported smell and taste dysfunction, respectively. Table 1 shows the distribution of blood cadmium concentrations and demographic characteristics by self-reported taste and smell dysfunction status. Overall, individuals who reported taste and smell dysfunction had higher concentrations of blood cadmium than those without perceived dysfunction. Individuals with perceived taste (21.4% vs. 18.6%) and smell (23.6% vs. 17.9%) dysfunction were more likely to be aged between 70 and 80 years old when compared with those without perceived dysfunction. In addition, those with perceived smell dysfunction were more likely to be male (52.1% vs. 46.1%) or non-Hispanic White (77.8% vs. 70.7%), while those with perceived taste dysfunction were more likely to be female (55.8% vs. 52.1%) or Hispanic (14.8% vs. 10.4%). Moreover, individuals with perceived taste dysfunction had lower income and education level, while no difference in income and education was observed between those with and without perceived smell dysfunction. Furthermore, individuals with perceived taste or smell dysfunction were more likely to be current smokers, and those who with only perceived smell dysfunction were more likely to be obese or current drinkers.

Table 2 shows the distribution of blood cadmium concentrations and demographic characteristics by status of objectively measured taste and smell dysfunction. A total of 1703 individuals were included in the analyses of objectively measured taste and smell dysfunction, with 287 (16.9%) and 260 (17.3%) individuals identified as having smell and taste dysfunction, respectively. Consistently, individuals with objectively measured taste and smell dysfunction were more likely to be between 70 and 80 years old, less educated, or have lower income. Individuals with objectively measured smell dysfunction were more likely to be male, while those with objectively measured taste dysfunction were more likely to be female. No difference was observed for race/ethnicity, BMI, and alcohol drinking, except that individuals with objectively measured taste dysfunction were more likely to be Hispanic, obese, or current smokers.

Table 3 shows the associations between blood cadmium and perceived, objectively measured, and codefined taste and smell dysfunction. Blood cadmium was analyzed both as a continuous variable (log-transformed cadmium) and a categorical variable (tertiles). In the crude-adjusted model controlling for age, gender, race/ethnicity, education, and PIR, higher blood cadmium level was significantly associated with perceived smell dysfunction (OR = 1.16, 95% CI: 1.01, 1.33) and perceived taste dysfunction (OR = 1.19, 95% CI: 1.05, 1.34). Consistent results were observed from the models with blood cadmium analyzed as a categorical variable. Significantly higher odds of having perceived smell dysfunction (OR = 1.41, 95% CI: 1.08, 1.84) and perceived taste dysfunction (OR = 1.48, 95% CI: 1.16, 1.89) were observed among individuals with a blood cadmium level in the highest tertile. In the fully adjusted models which further controlled for BMI, cigarette smoking, and alcohol drinking, consistent results were observed for perceived taste dysfunction among individuals with blood cadmium in the highest tertile (OR = 1.49, 95% CI: 1.10, 2.00), as well as in the model where blood cadmium was treated as a continuous variable (OR = 1.24, 95% CI: 1.07, 1.43). No statistically significant association was found between blood cadmium and perceived smell dysfunction. In addition, no statistically significant association was observed between blood cadmium and objectively measured taste and smell dysfunction. Significant associations were observed between blood cadmium and taste and smell dysfunction determined by both the questionnaires and examinations. Individuals with a blood cadmium level in the highest tertile had significantly higher odds of taste dysfunction (determined by both the questionnaires and examinations) in the crude-adjusted model (OR = 1.46, 95% CI: 1.03, 2.07). However, the associations were no longer significant after further controlling for BMI, smoking, and alcohol drinking. Higher odds of smell dysfunction (determined by both the questionnaires and examinations) were also observed among individuals with a cadmium level in the highest tertile. Nevertheless, none of these associations were statistically significant.

Table 4 shows the correlations between taste dysfunction and smell dysfunction defined by questionnaires, examinations, and both criteria. Statistically significant correlations between perceived and codefined taste and smell dysfunction were observed. However, no significant correlation was observed between measured taste and smell dysfunction.

## 4. Discussion

Our study showed significant associations between cadmium exposure and perceived taste and smell dysfunction. The association remained significant only for perceived taste dysfunction in the fully adjusted model after controlling for additional confounders. Significant associations between blood cadmium and taste dysfunction defined by both questionnaires and examinations were observed only in the crude-adjusted models.

Our findings were consistent with previous animal studies and population studies in occupational settings. In a mouse study, Bondier et al. [35] found that the olfactory route was likely to be one way by which cadmium can reach the brain. Sulkowski et al. [36] found significant associations between olfactory dysfunction and blood and urinary cadmium levels. In our study, no significant association was observed between blood cadmium and objectively measured taste and smell dysfunction. There are several potential reasons, including (1) the exclusion of individuals who were unable to pass the gLMS test along with those who had poor or fair understanding of the test, and (2) the proportions of individuals with blood cadmium concentrations below LLOD differed between individuals with and without measured taste dysfunction, which might introduce differential exposure misclassifications and bias the results towards the null. Although no significant association was found for smell dysfunction, the point estimate observed was consistent with taste dysfunction, suggesting significant associations might be observed with a larger sample size. We also found that perceived taste dysfunction was significantly correlated with perceived smell dysfunction, suggesting people with smell dysfunction were also likely to have taste dysfunction. Significant correlations were also observed between codefined taste and smell dysfunction. However, the strengths of the correlations were relatively small and no correlation was observed between objectively measured taste and smell dysfunction. Consistently, a previous study examined taste and smell functions based on objective measures and found severe taste dysfunction had higher prevalence than severe smell dysfunction among older adults [37]. In addition, when compared with the results from Lee et al. [31], we observed a smaller effect size of cadmium exposure. The difference might have been caused by differences in (1) the study population—we used a nationally representative population in this study, while a high-risk population (i.e., workers from certain types of industries) was targeted by Lee et al. [31]—and (2) the exposure measurement—cadmium was assessed using blood samples in our study.

However, several limitations need to be noted when interpreting the results from this study. First, a cross-sectional design was used in this study, where temporality and causality between the exposure and outcomes could not be well established. It is possible that taste dysfunction might lead to higher exposure to cadmium because of the lack of ability to detect hazards. Second, individuals who failed the gLMS tests were excluded from the taste tests, which may introduce potential selection bias. Recall bias could not be avoided since perceived taste and smell dysfunction was determined by self-reported information. Even though the questions were selected based on prior reliability and validity studies, misclassification of the outcomes may still exist. Third, cadmium exposure was measured using blood samples, which reflect the biologically effective dose at the time when individuals were tested and may not represent cadmium exposure in the past. Additionally, around 10% of the individuals included in this study had blood cadmium concentrations below the LLOD. Lastly, there might be other coexposed pollutants which may act independently or interactively with cadmium on taste and smell dysfunction.

Our study has several strengths. To our knowledge, this is the first study investigating the associations between cadmium exposure and taste and smell dysfunction in a nationally representative population. Most previous studies assessed taste and smell dysfunction based on only self-reported data. In our study, we combined objective measures with self-reported information to define both smell and taste dysfunction. Findings from Mascagni et al. [38] suggest that olfactory tests could be used to detect early effects of xenobiotics even at a low exposure level. The significant associations we observed between cadmium exposure and taste dysfunction bridged the gaps and highlighted the potential role of cadmium exposure in the development of taste dysfunction.

## 5. Conclusions

In conclusion, blood cadmium was observed to be significantly associated with perceived taste dysfunction among adults aged 40–80 years old in the United States. Further studies are warranted to confirm this finding and to explore and understand the potential underlying mechanisms.

## Figures and Tables

**Table 1:** Distributions of characteristics by perceived taste and smell dysfunction among adults in the National Health and Nutrition Examination Survey (NHANES) 2011–2014 (*n* = 5038).

Characteristics	Perceived Smell Dysfunction				Perceived Taste Dysfunction			
	Yes (*n* = 907)		No (*n* = 4124)		Yes (*n* = 771)		No (4236)	
	*n*	Mean ± SD ^a^/ Percent (95% CI) ^b^	*n*	Mean ± SD ^a^ / Percent (95% CI) ^b^	*n*	Mean ± SD ^a^ / Percent (95% CI) ^b^	*n*	Mean ± SD ^a^ / Percent (95% CI) ^b^
Blood cadmium (μg/L)	907	0.39 ± 0.04	4124	0.35 ± 0.02	771	0.41 ± 0.03	4236	0.35 ± 0.02
Blood cadmium < LLOD ^c^	75	10.4 (6.7–15.7)	308	9.9 (8.2–11.9)	57	9.7 (7.0–13.4)	324	10.0 (8.3–12.1)
Age (years)								
40–49	186	25.9 (21.9–30.3)	1135	30.5 (28.3–32.7)	174	26.0 (19.9–33.4)	1141	30.2 (27.9–32.6)
50–59	219	28.4 (25.0–32.0)	1062	29.9 (27.8–32.2)	192	31.2 (26.0–37.0)	1085	29.5 (27.5–31.5)
60–69	239	22.2 (19.3–25.4)	1023	21.6 (19.6–23.8)	212	21.3 (17.9–25.1)	1041	21.7 (19.9–23.7)
70–80	263	23.6 (20.4–27.1)	904	17.9 (16.6–19.4)	193	21.4 (17.4–26.1)	969	18.6 (17.2–20.1)
Gender								
Male	471	52.1 (47.9–56.3)	1957	46.1 (44.1–48.2)	334	44.2 (40.3–48.0)	2078	47.8 (45.8–49.7)
Female	436	47.9 (43.7–52.1)	2167	53.9 (51.8–55.9)	437	55.8 (52.0–59.7)	2158	52.2 (50.3–54.2)
Race/ethnicity								
Non-Hispanic White	460	77.8 (71.9–82.8)	1590	70.7 (64.8–76.0)	308	68.5 (62.1–74.2)	1737	72.8 (67.1–77.9)
Non-Hispanic Black	175	7.2 (5.0–10.4)	1048	10.8 (7.7–14.8)	183	11.1 (7.6–15.9)	1031	9.9 (7.2–13.5)
Hispanic	172	9.2 (6.2–13.4)	860	11.4 (8.6–15.0)	202	14.8 (11.0–19.6)	826	10.4 (7.7–13.8)
Others	100	5.8 (4.0–8.2)	626	7.1 (5.7–8.7)	78	5.7 (3.8–8.5)	642	6.9 (5.7–8.4)
IPR ^c^								
<1.0	197	13.5 (11.1–16.4)	770	11.2 (9.2–13.7)	216	18.6 (15.4–22.4)	746	10.5 (8.6–12.9)
1.0-2.0	240	19.5 (15.3–24.4)	970	18.9 (16.1–22.1)	200	24.5 (20.3–29.3)	1001	18.1 (15.5–21.2)
≥2.0	408	61.8 (54.7–68.4)	2002	62.9 (58.8–66.9)	293	51.0 (45.7–56.3)	2110	64.6 (60.2–68.8)
Missing	62	5.3 (3.3–8.2)	382	6.9 (5.7–8.3)	62	5.9 (4.2–8.1)	379	6.7 (5.5–8.21)
Education								
<high school	216	15.5 (11.9–19.8)	1084	17.3 (14.8–20.3)	259	23.2 (19.4–27.6)	1025	15.9 (13.4–18.7)
high school	204	21.9 (17.5–27.1)	912	21.7 (19.5–24.1)	162	20.8 (16.4–26.1)	953	22.0 (19.6–24.7)
>high school	487	62.6 (56.2–68.6)	2126	60.9 (57.1–64.6)	350	55.9 (50.4–61.3)	2255	62.1 (58.1–65.9)
Missing	0	0.0 (0.0–0.0)	2	0.0 (0.0–0.1)	0	0.0 (0.0–0.0)	3	0.0 (0.0–0.1)
BMI ^c^								
Underweight	15	1.5 (1.0–2.3)	56	1.0 (0.7–1.5)	13	1.2 (0.7–1.9)	58	1.1 (0.8–1.6)
Normal	220	24.8 (21.4–28.5)	1053	24.0 (22.4–25.7)	163	20.0 (16.4–24.2)	1107	24.9 (23.4–26.5)
Overweight	307	33.2 (28.9–37.9)	1387	36.3 (34.3–38.2)	239	32.3 (27.4–37.6)	1444	36.2 (34.5–37.9)
Obese	347	39.2 (34.6–44.0)	1570	37.6 (34.9–40.4)	339	43.9 (38.9–49.1)	1569	36.9 (34.3–39.5)
Missing	18	1.3 (0.8–2.2)	58	1.1 (0.8–1.5)	17	2.6 (1.5–4.4)	58	0.9 (0.7–1.2)
Smoking status								
Current smoker	201	21.2 (17.7–25.2)	702	16.8 (15.1–18.6)	177	21.9 (18.0–26.4)	722	16.9 (15.3–18.7)
Former smoker	296	33.5 (29.4–38.0)	1149	29.7 (27.5–32.0)	219	29.8 (24.9–35.3)	1219	30.6 (28.4–32.8)
Never	409	45.2 (40.5–50.0)	2270	53.5 (50.8–56.1)	374	48.2 (43.1–53.4)	2292	52.4 (49.7–55.1)
Missing	1	0.0 (0.0–0.3)	3	0.0 (0.0–0.1)	1	0.1 (0.0–0.4)	3	0.0 (0.0–0.1)
Alcohol drinking								
Current drinker	633	76.7 (72.7–80.2)	2575	71.3 (68.8–73.6)	488	69.9 (64.5–74.8)	2708	72.8 (70.3–75.2)
Former drinker	89	8.4 (6.6–10.7)	534	9.7 (8.6–10.9)	101	11.0 (7.8–15.3)	518	9.2 (8.1–10.4)
Never	114	8.6 (6.5–11.4)	619	11.1 (9.4–13.1)	122	12.4 (9.4–16.2)	605	10.3 (8.8–12.0)
Missing	71	6.3 (4.5–8.6)	396	7.8 (6.6–9.4)	60	6.6 (4.9–9.0)	405	7.7 (6.4–9.3)

^a^ Weighted geometric mean with standard error. ^b^ Weighted percentage with 95% confidence interval. ^c^ LLOD: lower limit of detection; IPR: income-to-poverty ratio; BMI: body mass index.

**Table 2:** Distributions of characteristics by objectively measured taste and smell dysfunction among adults in the NHANES 2011–2014 (*n* = 1703).

Characteristics	Measured Smell Dysfunction				Measured Taste Dysfunction			
	Yes (*n* = 287)		No (*n* = 1409)		Yes (*n* = 260)		No (*n* = 1242)	
	*n*	Mean ± SD ^a^ / Percent (95% CI) ^b^	*n*	Mean ± SD ^a^ / Percent (95% CI) ^b^	*n*	Mean ± SD ^a^ / Percent (95% CI) ^b^	*n*	Mean ± SD ^a^ / Percent (95% CI) ^b^
Blood cadmium (μg/L)		0.35 ± 0.04		0.33 ± 0.04		0.32 ± 0.06		0.33 ± 0.03
Blood cadmium < LLOD ^c^	16	4.4 (1.9–10.3)	34	4.2 (2.6–6.5)	10	8.3 (5.6–12.1)	42	3.7 (2.0–6.8)
Age (years)								
40–49	33	10.8 (7.4–15.5)	431	31.6 (28.4–34.9)	85	33.9 (27.2–41.3)	338	28.4 (25.5–31.4)
50–59	57	23.8 (17.6–31.3)	378	30.3 (27.4–33.4)	73	30.8 (22.6–40.4)	329	30.2 (26.5–34.3)
60–69	76	25.1 (18.8–32.7)	346	22.4 (19.1–26.0)	49	17.8 (12.9–24.0)	314	23.4 (20.0–27.2)
70–80	121	40.3 (32.2–48.9)	254	15.7 (13.2–18.7)	53	17.6 (11.4–26.1)	261	18.0 (15.3–21.0)
Gender								
Male	169	54.3 (45.0–63.3)	638	47.1 (44.5–49.6)	128	51.2 (46.4–55.9)	597	48.5 (45.8–51.2)
Female	118	45.7 (36.7–55.0)	771	52.9 (50.4–55.5)	132	48.8 (44.1–53.6)	645	51.5 (48.8–54.2)
Race/ethnicity								
Non-Hispanic White	127	68.8 (61.5–75.2)	639	72.7 (64.8–79.4)	123	72.2 (62.7–80.1)	580	74.1 (66.4–80.5)
Non-Hispanic Black	59	11.2 (7.3–17.0)	278	10.0 (7.1–13.9)	62	12.9 (8.4–19.4)	231	9.0 (6.2–12.9)
Hispanic	62	12.2 (7.6–19.0)	302	10.6 (6.8–16.3)	52	10.1 (5.6–17.6)	258	10.1 (6.5–15.5)
Others	39	7.8 (4.8–12.6)	190	6.7 (5.0–8.9)	23	4.8 (3.2–7.1)	173	6.7 (5.0–9.0)
IPR ^c^								
<1.0	45	10.3 (6.3–16.3)	261	11.1 (8.0–15.3)	50	11.0 (6.2–18.6)	203	10.0 (7.0–14.2)
1.0-2.0	84	23.9 (18.0–31.1)	311	18.5 (14.9–22.7)	57	17.2 (12.6–22.9)	293	19.3 (15.3–24.0)
≥2.0	135	58.2 (48.7–67.1)	728	64.9 (58.1–71.2)	137	65.9 (58.6–72.6)	647	65.1 (58.0–71.6)
Missing	23	7.6 (4.3–13.3)	109	5.5 (3.8–7.9)	16	5.9 (2.7–12.5)	99	5.7 (4.1–7.8)
Education								
<high school	87	19.9 (13.8–27.8)	290	13.4 (10.1–17.7)	50	12.4 (8.0–18.9)	252	13.1 (9.8–17.3)
high school	64	20.6 (16.2–25.7)	319	22.7 (19.5–26.2)	70	26.7 (21.4–32.9)	271	21.2 (18.0–24.8)
>high school	135	59.4 (52.3–66.2)	800	63.9 (58.7–68.8)	140	60.8 (51.4–69.5)	719	65.7 (60.3–70.8)
Missing	1	0.1 (0.0–0.9)	0	0.0 (0.0–0.0)	0	0.0 (0.0–0.0)	0	0.0 (0.0–0.0)
BMI ^c^								
Underweight	4	0.8 (0.3–2.4)	24	1.2 (0.6–2.4)	5	1.7 (0.6–4.3)	20	1.0 (0.5–2.3)
Normal	74	22.8 (17.8–28.6)	357	24.0 (21.9–26.4)	63	25.2 (18.7–32.9)	316	23.1 (21.4–25.0)
Overweight	102	34.2 (27.0–42.2)	465	35.4 (32.4–38.5)	77	32.3 (23.7–42.3)	424	35.9 (31.8–40.2)
Obese	104	41.4 (34.5–48.8)	551	38.8 (35.0–42.8)	112	40.2 (31.9–49.2)	475	39.6 (35.3–44.1)
Missing	3	0.8 (0.2–2.8)	12	0.6 (0.2–1.3)	3	0.6 (0.2–2.0)	7	0.3 (0.1–0.8)
Smoking status								
Current smoker	45	13.0 (8.9–18.5)	249	15.7 (12.3–19.7)	49	15.1 (10.3–21.7)	208	15.2 (12.5–18.3)
Former smoker	100	40.7 (29.5–52.8)	383	29.8 (25.4–34.6)	81	36.0 (27.4–45.6)	362	31.5 (27.7–35.5)
Never	142	46.4 (36.7–56.3)	777	54.6 (48.7–60.3)	130	48.8 (41.3–56.4)	672	53.4 (47.7–59.0)
Missing	0	0.0 (0.0–0.0)	0	0.0 (0.0–0.0)	0	0.0 (0.0–0.0)	0	0.0 (0.0–0.0)
Alcohol drinking								
Current drinker	160	64.7 (54.0–74.1)	960	75.8 (71.1–79.9)	187	78.1 (70.0–84.5)	841	75.7 (69.6–81.0)
Former drinker	40	12.7 (8.2–19.2)	174	8.9 (6.7–11.7)	35	9.2 (5.7–14.5)	158	9.4 (6.7–12.9)
Never	62	15.7 (10.2–23.6)	182	10.1 (7.4–13.6)	23	8.1 (3.7–16.8)	168	10.0 (7.1–13.8)
Missing	25	6.8 (3.7–12.3)	93	5.2 (3.9–7.0)	15	4.6 (3.0–7.1)	75	4.9 (3.9–6.3)

^a^ Weighted geometric mean with standard error; ^b^ Weighted percentage with 95% confidence interval. ^c^ LLOD: lower limit of detection; IPR: income-to-poverty ratio; BMI: body mass index.

**Table 3:** Associations between blood cadmium and taste and smell dysfunction among adults in the NHANES 2011-2014.

Outcomes	Log-Transformed Cadmium Levels	
	Crude-adjusted ^a^	Fully adjusted ^b^
	OR (95% CI) ^c^	OR (95% CI) ^c^
Perceived smell dysfunction (*n* = 5031)		
Continuous	1.16 (1.01, 1.33)	1.05 (0.88, 1.26)
Categorical		
Tertile 1	Reference	Reference
Tertile 2	1.15 (0.84, 1.56)	1.10 (0.81, 1.49)
Tertile 3	1.41 (1.08, 1.84)	1.24 (0.92, 1.65)
Perceived taste dysfunction (*n* = 5007)		
Continuous	1.19 (1.05, 1.34)	1.24 (1.07, 1.43)
Categorical		
Tertile 1	Reference	Reference
Tertile 2	1.28 (0.96, 1.69)	1.30 (0.96, 1.76)
Tertile 3	1.48 (1.16, 1.89)	1.49 (1.10, 2.00)
Measured smell dysfunction (*n* = 1696)		
Continuous	1.06 (0.87, 1.30)	0.98 (0.73, 1.30)
Categorical		
Tertile 1	Reference	Reference
Tertile 2	1.26 (0.77, 2.06)	1.17 (0.73, 1.89)
Tertile 3	1.27 (0.83, 1.95)	1.16 (0.70, 1.95)
Measured taste dysfunction (*n* = 1502)		
Continuous	0.97 (0.79, 1.19)	0.93 (0.69, 1.25)
Categorical		
Tertile 1	Reference	Reference
Tertile 2	0.94 (0.59, 1.48)	0.92 (0.58, 1.47)
Tertile 3	1.09 (0.73, 1.64)	1.08 (0.61, 1.93)
Codefined smell dysfunction (*n* = 1696)		
Continuous	1.08 (0.88, 1.33)	0.93 (0.71, 1.20)
Categorical		
Tertile 1	Reference	Reference
Tertile 2	1.17 (0.63, 2.17)	1.08 (0.60, 1.97)
Tertile 3	1.35 (0.76, 2.41)	1.15 (0.61, 2.19)
Codefined taste dysfunction (*n* = 1502)		
Continuous	1.11 (0.97, 1.25)	1.08 (0.87, 1.34)
Categorical		
Tertile 1	Reference	Reference
Tertile 2	1.15 (0.81, 1.65)	1.13 (0.79, 1.61)
Tertile 3	1.46 (1.03, 2.07)	1.47 (0.85, 2.52)

^a^ Crude-adjusted model: adjusting for age, gender, race/ethnicity, education, and income-to-poverty ratio (IPR). ^b^ Fully adjusted model: adjusting for age, gender, race/ethnicity, education, IPR, body mass index (BMI), cigarette smoking, and alcohol drinking. ^c^ Weighted odds ratio with 95% confidence interval.

**Table 4:** Correlations between taste dysfunction and smell dysfunction.

Outcomes	Correlation ^a^	Standard Error	*t* value	*p* Value
Perceived taste and smell dysfunction	0.24	0.01	17.3	<0.001
Measured taste and smell dysfunction	0.04	0.03	1.48	0.14
Codefined taste and smell dysfunction	0.13	0.03	5.10	<0.001

^a^ Weighted Pearson’s correlation coefficient.

## Supplementary Materials

The following are available online at https://www.mdpi.com/1660-4601/17/3/943/s1, Figure S1: Directed acyclic graphs (DAGs) for selecting minimized sets of variables of the crude-adjusted models (A) and the fully-adjusted models (B).

## References

[1] Hummel T., Croy I., Haehner A. (2016). Olfactory disorders and consequences. Flavor.

[2] Keller A., Malaspina D. (2013). Hidden consequences of olfactory dysfunction: A patient report series. BMC Ear Nose Throat Disord..

[3] Kamath V., Paksarian D., Cui L., Moberg P.J., Turetsky B.I., Merikangas K.R. (2018). Olfactory processing in bipolar disorder, major depression, and anxiety. Bipolar Disord..

[4] Negoias S., Hummel T., Symmank A., Schellong J., Joraschky P., Croy I. (2016). Olfactory bulb volume predicts therapeutic outcome in major depression disorder. Brain Imaging Behav..

[5] Taalman H., Wallace C., Milev R. (2017). Olfactory Functioning and Depression: A Systematic Review. Front. Psychiatry.

[6] Hur K., Choi J.S., Zheng M., Shen J., Wrobel B. (2018). Association of alterations in smell and taste with depression in older adults. Laryngoscope Investig Otolaryngol.

[7] Schäfer L., Mehler L., Hähner A., Walliczek U., Hummel T., Croy I. (2018). Sexual desire after olfactory loss: Quantitative and qualitative reports of patients with smell disorders. Physiol. Behav..

[8] Seubert J., Laukka E.J., Rizzuto D., Hummel T., Fratiglioni L., Bäckman L., Larsson M. (2017). Prevalence and Correlates of Olfactory Dysfunction in Old Age: A Population-Based Study. J. Gerontol. A Biol. Sci. Med. Sci..

[9] Sjölund S., Larsson M., Olofsson J.K., Seubert J., Laukka E.J. (2017). Phantom Smells: Prevalence and Correlates in a Population-Based Sample of Older Adults. Chem. Senses.

[10] Temmel A.F.P., Quint C., Schickinger-Fischer B., Klimek L., Stoller E., Hummel T. (2002). Characteristics of olfactory disorders in relation to major causes of olfactory loss. Arch. Otolaryngol. Head. Neck Surg..

[11] Wang T., Glendinning J., Grushka M., Hummel T., Mansfield K. (2017). From the Cover: Drug-Induced Taste Disorders in Clinical Practice and Preclinical Safety Evaluation. Toxicol. Sci..

[12] Brion M., de Timary P., Vander Stappen C., Guettat L., Lecomte B., Rombaux P., Maurage P. (2015). Chemosensory Dysfunction in Alcohol-Related Disorders: A Joint Exploration of Olfaction and Taste. Chem. Senses.

[13] Uecker F.C., Olze H., Kunte H., Gerz C., Göktas Ö., Harms L., Schmidt F.A. (2017). Longitudinal Testing of Olfactory and Gustatory Function in Patients with Multiple Sclerosis. PLoS ONE.

[14] Kouzuki M., Suzuki T., Nagano M., Nakamura S., Katsumata Y., Takamura A., Urakami K. (2017). Comparison of olfactory and gustatory disorders in Alzheimer’s disease. Neurol. Sci..

[15] Iannilli E., Stephan L., Hummel T., Reichmann H., Haehner A. (2017). Olfactory impairment in Parkinson’s disease is a consequence of central nervous system decline. J. Neurol..

[16] Cohen J., Wakefield C.E., Laing D.G. (2016). Smell and Taste Disorders Resulting from Cancer and Chemotherapy. Curr. Pharm. Des..

[17] Belqaid K., Tishelman C., McGreevy J., Månsson-Brahme E., Orrevall Y., Wismer W., Bernhardson B.-M. (2016). A longitudinal study of changing characteristics of self-reported taste and smell alterations in patients treated for lung cancer. Eur. J. Oncol. Nurs..

[18] Liu G., Zong G., Doty R.L., Sun Q. (2016). Prevalence and risk factors of taste and smell impairment in a nationwide representative sample of the US population: A cross-sectional study. BMJ Open.

[19] Rawal S., Hoffman H.J., Bainbridge K.E., Huedo-Medina T.B., Duffy V.B. (2016). Prevalence and Risk Factors of Self-Reported Smell and Taste Alterations: Results from the 2011-2012 US National Health and Nutrition Examination Survey (NHANES). Chem. Senses.

[20] Ajmani G.S., Suh H.H., Pinto J.M. (2016). Effects of Ambient Air Pollution Exposure on Olfaction: A Review. Environ. Health Perspect..

[21] Adams D.R., Ajmani G.S., Pun V.C., Wroblewski K.E., Kern D.W., Schumm L.P., McClintock M.K., Suh H.H., Pinto J.M. (2016). Nitrogen dioxide pollution exposure is associated with olfactory dysfunction in older U.S. adults. Int. Forum Allergy Rhinol..

[22] Wu H., Liao Q., Chillrud S.N., Yang Q., Huang L., Bi J., Yan B. (2016). Environmental Exposure to Cadmium: Health Risk Assessment and its Associations with Hypertension and Impaired Kidney Function. Sci. Rep..

[23] Song J.K., Luo H., Yin X.H., Huang G.L., Luo S.Y., Du ren L., Yuan D.B., Zhang W., Zhu J.G. (2015). Association between cadmium exposure and renal cancer risk: A meta-analysis of observational studies. Sci. Rep..

[24] Chen C., Xun P., Nishijo M., Carter S., He K. (2016). Cadmium exposure and risk of prostate cancer: A meta-analysis of cohort and case-control studies among the general and occupational populations. Sci. Rep..

[25] Kang M.-Y., Cho S.-H., Leem Y.-H., Kim J.-H., Bae S.-H., Hong Y.-C. (2011). Association between cadmium and cognitive function in the elderly. Korean J. Occup. Environ. Med..

[26] Hutton M. (1983). Sources of cadmium in the environment. Ecotoxicol. Environ. Saf..

[27] Pinot F., Kreps S.E., Bachelet M., Hainaut P., Bakonyi M., Polla B.S. (2000). Cadmium in the environment: Sources, mechanisms of biotoxicity, and biomarkers. Rev. Environ. Health.

[28] He P., Lu Y., Liang Y., Chen B., Wu M., Li S., He G., Jin T. (2013). Exposure assessment of dietary cadmium: Findings from Shanghainese over 40 years, China. BMC Public Health.

[29] Alloway B.J., Jackson A.P., Morgan H. (1990). The accumulation of cadmium by vegetables grown on soils contaminated from a variety of sources. Sci. Total Environ..

[30] Riederer A.M., Belova A., George B.J., Anastas P.T. (2013). Urinary cadmium in the 1999--2008 US national health and nutrition examination survey (NHANES). Environ. Sci. Technol..

[31] Lee S.-J., Kim E.-M., Cho S.-H., Song J., Jang T.-W., Lee M.-Y. (2018). Risk of olfactory dysfunction of the workers in the automobile repair, printing, shoemaking and plating industries in Korea: A cross-sectional study. BMJ Open.

[32] Rawal S., Hoffman H.J., Honda M., Huedo-Medin T.B., Duffy V.B. (2015). The Taste and Smell Protocol in the 2011-2014 US National Health and Nutrition Examination Survey (NHANES): Test-Retest Reliability and Validity Testing. Chemosens. Percept..

[33] Doty R.L., Shaman P., Dann M. (1984). Development of the university of pennsylvania smell identification test: A standardized microencapsulated test of olfactory function. Physiol. Behav..

[34] CDC Questionnaire, datasets, and related documentation. https://wwwn.cdc.gov/nchs/nhanes/.

[35] Bondier J.-R., Michel G., Propper A., Badot P.-M. (2008). Harmful effects of cadmium on olfactory system in mice. Inhal. Toxicol..

[36] Sulkowski W.J., Rydzewski B., Miarzynska M. (2000). Smell impairment in workers occupationally exposed to cadmium. Acta Otolaryngol..

[37] Boesveldt S., Lindau S.T., McClintock M.K., Hummel T., Lundstrom J.N. (2011). Gustatory and olfactory dysfunction in older adults: A national probability study. Rhinology.

[38] Mascagni P., Consonni D., Bregante G., Chiappino G., Toffoletto F. (2003). Olfactory function in workers exposed to moderate airborne cadmium levels. Neurotoxicology.

