# Supplementary material for: Associations between Cadmium Exposure and Taste and Smell Dysfunction: Results from the National Health and Nutrition Examination Survey (NHANES), 2011–2014
Source: Int J Environ Res Public Health. 2020 Feb 3;17(3):943. doi: 10.3390/ijerph17030943 (PMC7037909; doi:10.3390/ijerph17030943)
Supplement: Supplementary file 1 [file ijerph-17-00943-s001.pdf]

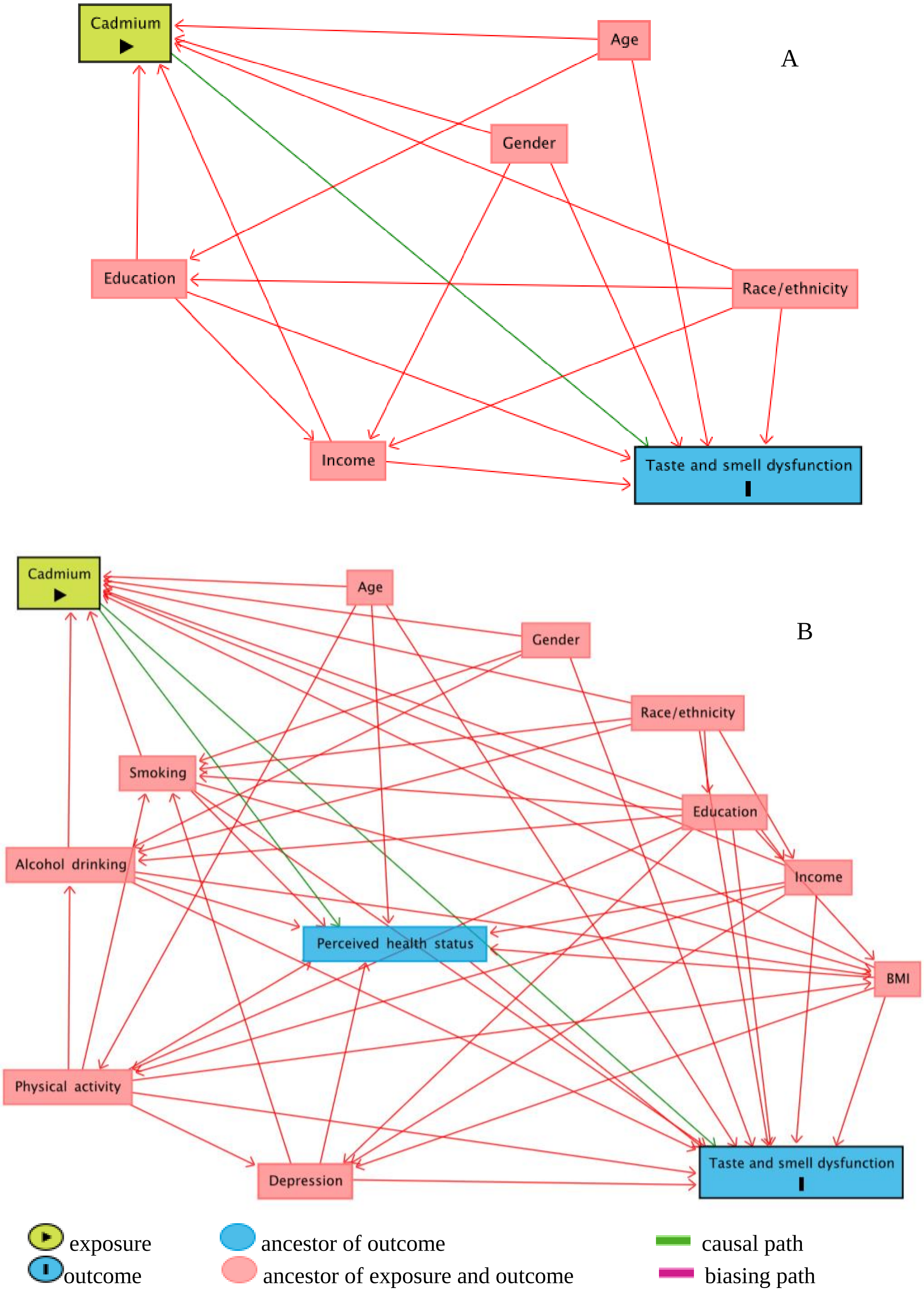

Supplemental Figure 1. Directed acyclic graphs (DAGs) for selecting minimized sets of variables of the crude-adjusted models (A) and the fully-adjusted models (B).
